# Impact of prior cancer history on the survival of patients with larynx cancer

**DOI:** 10.1186/s12885-020-07634-2

**Published:** 2020-11-23

**Authors:** Kaiquan Zhu, Renyu Lin, Ziheng Zhang, Huanqi Chen, Xingwang Rao

**Affiliations:** Department of otolaryngology, The First Affiliated Hospital of Wenzhou Medical University, Wenzhou, Zhejiang Province 325000 P. R. China

**Keywords:** Larynx cancer, Prior cancer, Survival, SEER, Trial eligibility

## Abstract

**Background:**

Patients with a prior history of cancer are commonly excluded from clinical trial. Increasing number of studies implied that a prior cancer did not adversely affect the clinical outcome among various types of cancer patients. However, the impact of prior cancer on survival of larynx cancer patients remains largely unknown. The aim of this study was to evaluate the prevalence of prior cancer and assess its impact on survival of patients diagnosed with larynx cancer.

**Methods:**

Patients with larynx cancer as the first or second primary malignancy diagnosed from 2004 to 2015 were extracted from the Surveillance, Epidemiology, and End Results (SEER) database. Propensity score matching (PSM) was conducted to balance baseline characteristics. Kaplan-Meier method, multivariate Cox proportional hazard model, and multivariate competing risk model were performed for survival analysis.

**Results:**

A total of 24,812 eligible patients with larynx cancer were included in the study, wherein a total of 2436 patients (9.8%) had a prior history of cancer. Prostate (36%), lung and bronchus (10%), urinary bladder (7%), and breast (6%) were the most common types of prior cancer. A prior cancer history served as a risk factor for overall survival (AHR =1.30; 95% CI [1.21–1.41]; *P* < 0.001) but a protective factor for cancer-specific mortality (AHR = 0.83; 95% CI [0.72–0.94]; *P* = 0.004) in comparison with those without prior cancer. The subgroup analysis showed that a prior history of cancer adversely affected overall survival of patients with larynx cancer in most subgroups stratified by timing and types of prior cancer, as well as by different clinicopathologic features.

**Conclusion:**

Our study indicated an adverse survival impact of a prior history of cancer on patients with larynx cancer. Except for a few particular prior cancer, clinical trials should be considered prudently for laryngeal cancer patients with prior cancers.

**Supplementary Information:**

The online version contains supplementary material available at 10.1186/s12885-020-07634-2.

## Background

Over the past thirty years, the population of cancer survivors is growing rapidly in the United States. A previous report revealed that there were three million cancer survivors in the United States in 1971, which, however, soared to a total of 11.7 million in the year 2007, indicating an approximately four-fold increase growth rate [1]. More importantly, the number has been on the rise partly due to the aging population and the significant advances made in the early detection and treatment of cancer [2, 3]. There were about two-thirds of cancer survivors who had survived for more than five years after the initial diagnosis [2], which caused an increased risk for developing multiple primary cancer [4–6].

Larynx cancer is the second most common cancer in the head and neck region, accounting for an estimated 13,150 new cases and a total of 3710 deaths in the United States [7]. Recently, along with the increased number of cancer survivors, larynx cancer was frequently diagnosed as the second primary malignant neoplasms (SPMs). Unfortunately, those patients with a prior cancer history were ruled out by most of the clinical trials concentrating on larynx cancer [8–12]. Given the sizable number of these patients, the exclusion criterion may limit the accuracy and generalizability of clinical trials, thus leaving some essential clinical questions unanswered [13, 14]. This stringent criterion is mainly due to concerns regarding prior treatment interference and its survival impact, though little evidence supports this assumption. Also, several previous retrospective studies reported that a prior cancer history did not adversely affect clinical outcomes of patients with various cancer [15–17]. However, since few studies have focused on the clinical outcome of patients with secondary laryngeal cancer, much is unknown as to if a prior cancer history impacts the clinical outcome of laryngeal cancer patients.

Therefore, to address this issue, the current study aimed to determine the impact of prior cancers on clinical outcomes among patients who developed larynx cancer as a second primary malignancy by using the Surveillance, Epidemiology, and End Results (SEER) database. We also hope the findings of this study may provide potential insight into the clinical management of laryngeal cancer patients with a prior cancer history.

## Methods

### Database and case selection

Data was obtained from the custom SEER database [Incidence- SEER 18 Regs Custom Data (with additional treatment fields), Nov 2018 Sub (1975–2016 varying)], which collected cancer data that covers approximately 28% of the United States population [18]. The SEER*Stat software version 8.3.6.1 (National Cancer Institute, USA) was utilized to access the data from SEER database. The patients who were diagnosed with larynx cancer (site code C32.0, C32.1, C32.2 C32.3, and C32.8) from 2004 to 2015 were extracted from the database. Only patients with only one primary tumor or patients who had exactly one prior malignancy were included. In order to exclude the synchronous primary cancers, a 2-month time interval was required between the prior cancer diagnosis and the second larynx cancer diagnosis. Other exclusion criteria were listed as follows: 1) patients with incomplete follow-up; 2) patients whose diagnosis was made at autopsy or based on a death certificate; 3) and patients who had unknown information of diagnosed months. Finally, a total of 24,812 eligible patients were included in this study. The detail flowchart of case selection can be seen in Fig. S1.

### Covariates

The analysis involved multiple variables including demographic characteristics (year of diagnosis, age at diagnosis, gender, race, and marital status), disease characteristics (histologic grade, AJCC stage, and prior cancer type), and treatment characteristics (surgery, radiotherapy and chemotherapy). Specially, the continuous variables, age at diagnosis, were transformed into categorical variables (< 45, 45–64, and > 64). Marital status including divorced, single, widowed, separated and domestic partner were classified into other status. The history of prior cancer was determined from the SEER sequence numbers code, which records the sequence of all malignancies occurred over the lifetime of the patient. Based on the recode of year of diagnosis and month of diagnosis, we calculated the time interval between two cancer record by subtracting the diagnosis date of the prior cancer from that of index larynx cancer. Vital status record were utilized to calculate the overall survival (OS). In addition, patients who died directly from larynx cancer were recorded as dead (attributable to this cancer) based on the code of cause-specific death classification in the SEER program. Alive or dead from a cause other than larynx cancer were considered as a competing risk event and censored at the time of the event. Hence, the SEER cause-specific death classification was utilized to calculate the larynx cancer-specific mortality (LCSM).

### Statistical analysis

Based on the SEER sequence number recode, we categorized the patients into two groups, including patients with primary larynx cancer (PLC) and patients having larynx cancer as the second primary malignancy (SLC). Descriptive statistic was utilized to summarize the demographic and clinical factors of these two groups of patients. Clinicopathologic characteristics between patients with or without prior cancer were compared using Pearson chi-square tests. The Log-rank test or Gray’s test was used to compare differences of OS and LCSM between patients with or without prior cancer record before and after propensity score matching (PSM). The detailed description for PSM could be seen in Supplementary material. The multivariate Cox regression analysis was used to identify whether the prior cancer history was an independent prognostic factor for OS. Meanwhile, multivariable Fine and Gray competing-risk regression model was built to analyze the impact of prior cancer history on LCSM. The common demographic and clinical characteristics, including age, gender, race, marital status, AJCC stage (sixth edition), grade, and treatment modalities (surgery, chemotherapy, and radiotherapy) were included as covariates.

Descriptive statistic, Pearson Chi-square test, and Cox proportional hazards model were performed using SPSS 24.0 (IBM Corp). Kaplan-Meier plot, log-rank test, and the Fine and Gray competing-risk regression model were plotted or conducted by using R software version 4.0.0.

## Results

### Baseline characteristics

A total of 24,812 eligible patients with larynx cancer were included, of whom 2436 (9.8%) had a history of prior cancer. 67.3% of the patients had their prior cancer identified as AJCC stage I-II. The median time interval (IQR) between the index larynx cancer and the prior cancer was 63 (29–112) months, and the median follow-up (IQR) was 26 (11–58), starting from the diagnosis of larynx cancer (Table 1). After adjusted for propensity scores, all variables were well-balanced between patients with or without prior cancer (Table 2).

**Table 1:** Summary description of demographic and clinical factors (*N* = 2436)

At prior cancer diagnosis		At larynx cancer diagnosis	
Variable	Value	Variable	Value
Age, years		Age, years	
Mean	64.7	Mean	71.3
Median (IQR)	66 (58–72)	Median (IQR)	72 (64–79)
AJCC stage, n (%)		AJCC stage, n (%)	
I-II	803 (67.3)	I-II	1321 (54.2)
III-IV	263 (10.8)	III-IV	930 (38.2)
Unknown	1370 (21.9)	Unknown	185 (7.6)
Interval between diagnoses, months		Follow up from diagnosis of larynx cancer, months	
Mean	79.6	Mean	38.5
Median (IQR)	63 (29–112)	Median (IQR)	26 (11–58)

*IQR* interquartile range

**Table 2:** Baseline characteristics of patients with larynx cancer in the original/matched data sets (N = 24,812)

Characteristics	Original data set			Matched data set		
	No prior cancer *N* = 22,376 (%)	With prior cancer N = 2436 (%)	*P* value	No prior cancer N = 2436 (%)	With prior cancer N = 2436 (%)	P value
Age (years), mean (SD)	63.6 (11.6)	71.3 (10.1)	<.001	68.4 (10.0)	71.3 (10.1)	0.025
Year of diagnose(%)			0.001			0.643
2004–2009	10,484 (46.9)	1056 (43.3)		1040 (42.7)	1056 (43.3)	
2010–2015	11,892 (53.1)	1380 (56.7)		1396 (57.3)	1380 (56.7)	
Race			0.025			0.069
White	17,878 (79.9)	1981 (81.3)		2019 (82.9)	1981 (81.3)	
Black	3495 (15.6)	374 (15.4)		322 (13.2)	374 (15.4)	
Others/Unknown	1003 (4.5)	81 (3.3)		95 (3.9)	81 (3.3)	
Gender			0.032			0.964
Male	18,032 (80.6)	2007 (82.4)		2028 (83.3)	2007 (82.4)	
Female	4344 (19.4)	429 (17.6)		408 (16.7)	429 (17.6)	
Marital status			<.001			0.158
Single	4511 (20.2)	330 (13.5)		366 (15.0)	330 (13.5)	
Married	11,093 (49.6)	1309 (53.7)		1260 (51.7)	1309 (53.7)	
Others status ^a^	5591 (25.0)	641 (26.3)		675 (27.7)	641 (26.3)	
Unknown	1181 (5.3)	156 (6.4)		135 (5.5)	156 (6.4)	
Grade			0.546			0.170
Grade I	2992 (13.4)	319 (13.1)		323 (13.3)	319 (13.1)	
Grade II	10,175 (45.5)	1071 (44.0)		1082 (44.4)	1071 (44.0)	
Grade III	3919 (17.5)	448 (18.4)		387 (15.9)	448 (18.4)	
Grade IV	164 (0.7)	17 (0.7)		21 (0.9)	17 (0.7)	
Unknown	5126 (22.9)	581 (23.9)		623 (25.6)	581 (23.9)	
AJCC stage			<.001			0.402
I	7257 (32.4)	974 (40.0)		971 (39.9)	974 (40.0)	
II	3411 (15.2)	347 (14.2)		392 (16.1)	347 (14.2)	
III	3616 (16.2)	393 (16.1)		365 (15.0)	393 (16.1)	
IV	6529 (29.2)	537 (22.0)		532 (21.8)	537 (22.0)	
Unknown	1563 (7.0)	185 (7.6)		176 (7.2)	185 (7.6)	
Surgery			0.012			0.460
No/unknown	14,355 (64.2)	1500 (61.6)		1525 (62.6)	1500 (61.6)	
Yes	8021 (35.8)	936 (38.4)		911 (37.4)	936 (38.4)	
Chemotherapy			<.001			0.413
No/unknown	14,809 (66.2)	1868 (76.7)		804 (33.0)	831 (34.1)	
Yes	7567 (33.8)	568 (23.3)		1632 (67.0)	1605 (65.9)	
Radiotherapy			<.001			0.683
No/unknown	5723 (25.6)	831 (34.1)		1880 (77.2)	1868 (76.7)	
Yes	16,653 (74.4)	1605 (65.9)		556 (22.8)	568 (23.3)	

^a^ other status including divorced, widowed, separated or domestic partner

**Fig. 1:**
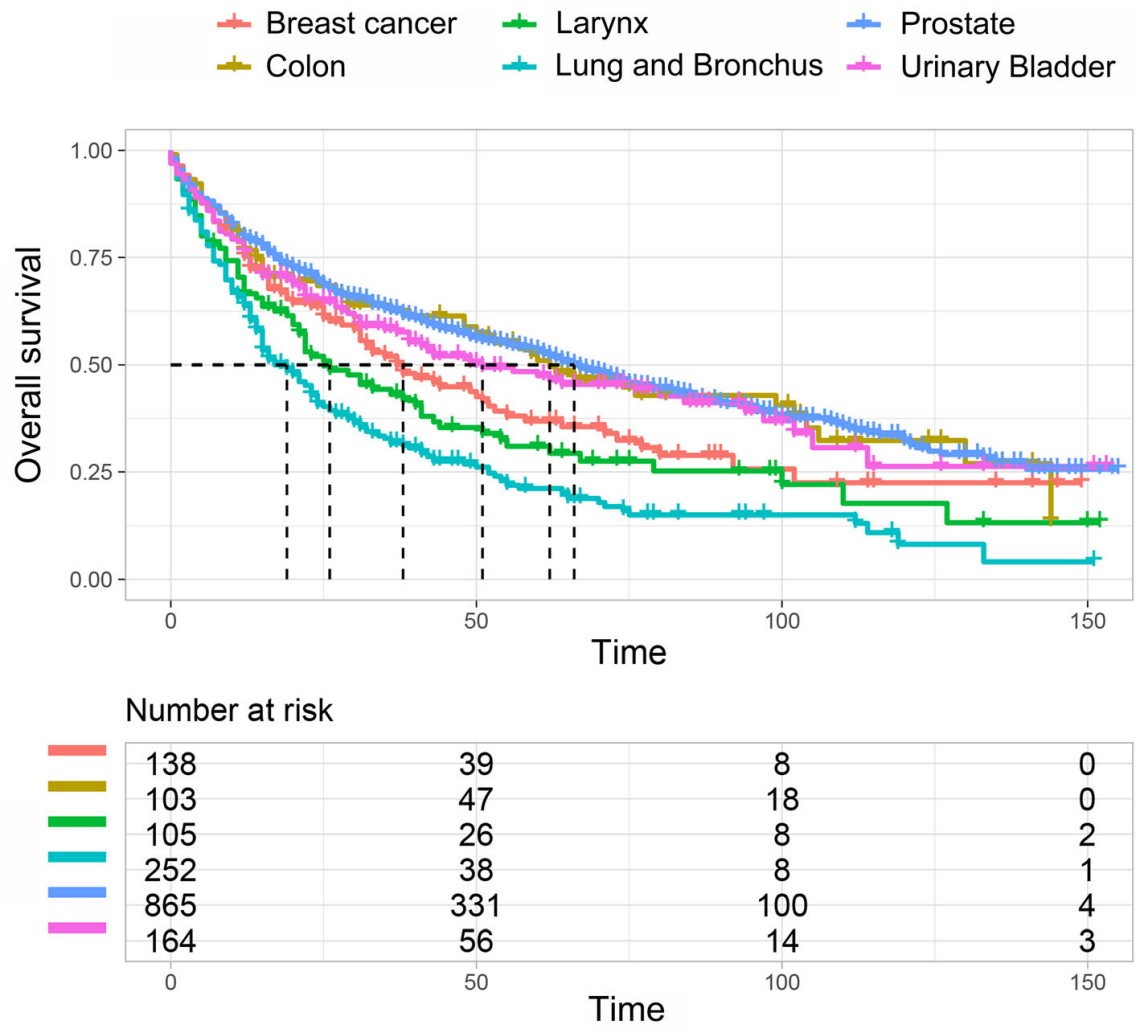
The overall survival (OS) of larynx cancer patient with different types of prior cancer

Prostate (36%), lung and bronchus (10%), urinary bladder (7%), and breast (6%) were the most common prior cancer types (Table 3). The overall survival (OS) varied significantly among larynx cancer patients who had different types of prior cancer (*P* < 0.001). Patients with prior lung cancer had the shortest OS, whereas those with prior prostate or colon cancer had a relatively good prognosis (Fig. 1).

**Table 3:** Distributions of prior cancer types

Prior cancer type	Number	Proportion (%)
Prostate	865	35.5%
Lung and Bronchus	252	10.3%
Urinary Bladder	164	6.7%
Breast	138	5.7%
Larynx	105	4.3%
Colon	103	4.2%
Kidney and Renal pelvis	65	2.7%
Tongue	64	2.6%
Melanoma of the skin	59	2.4%
Others site	621	25.5%

### Impact of prior cancer history on the survival of larynx cancer patients

**Fig. 2:**
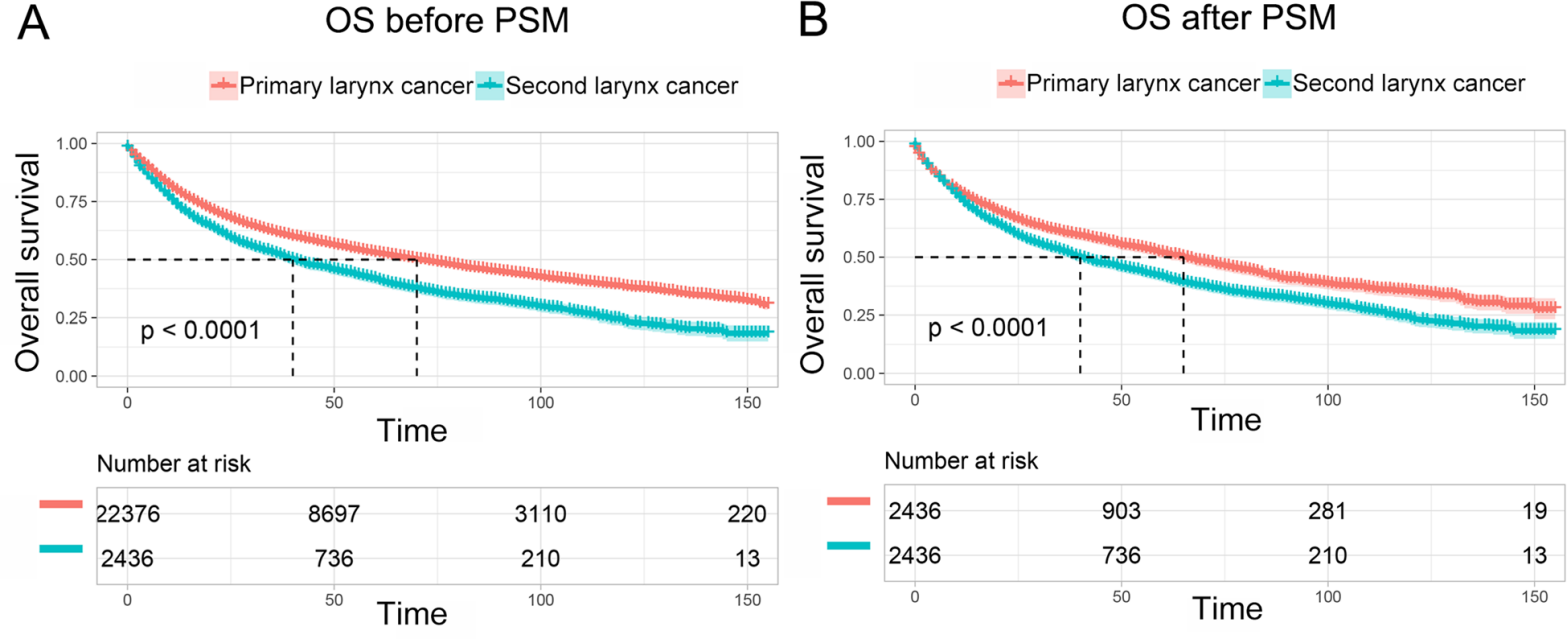
Kaplan-Meier survival curves of prior cancer impact on the overall survival (OS) in larynx cancer patients with or without prior cancer. **a** The OS analysis before Propensity score matching (PSM); **b** The OS analysis after PSM

**Fig. 3:**
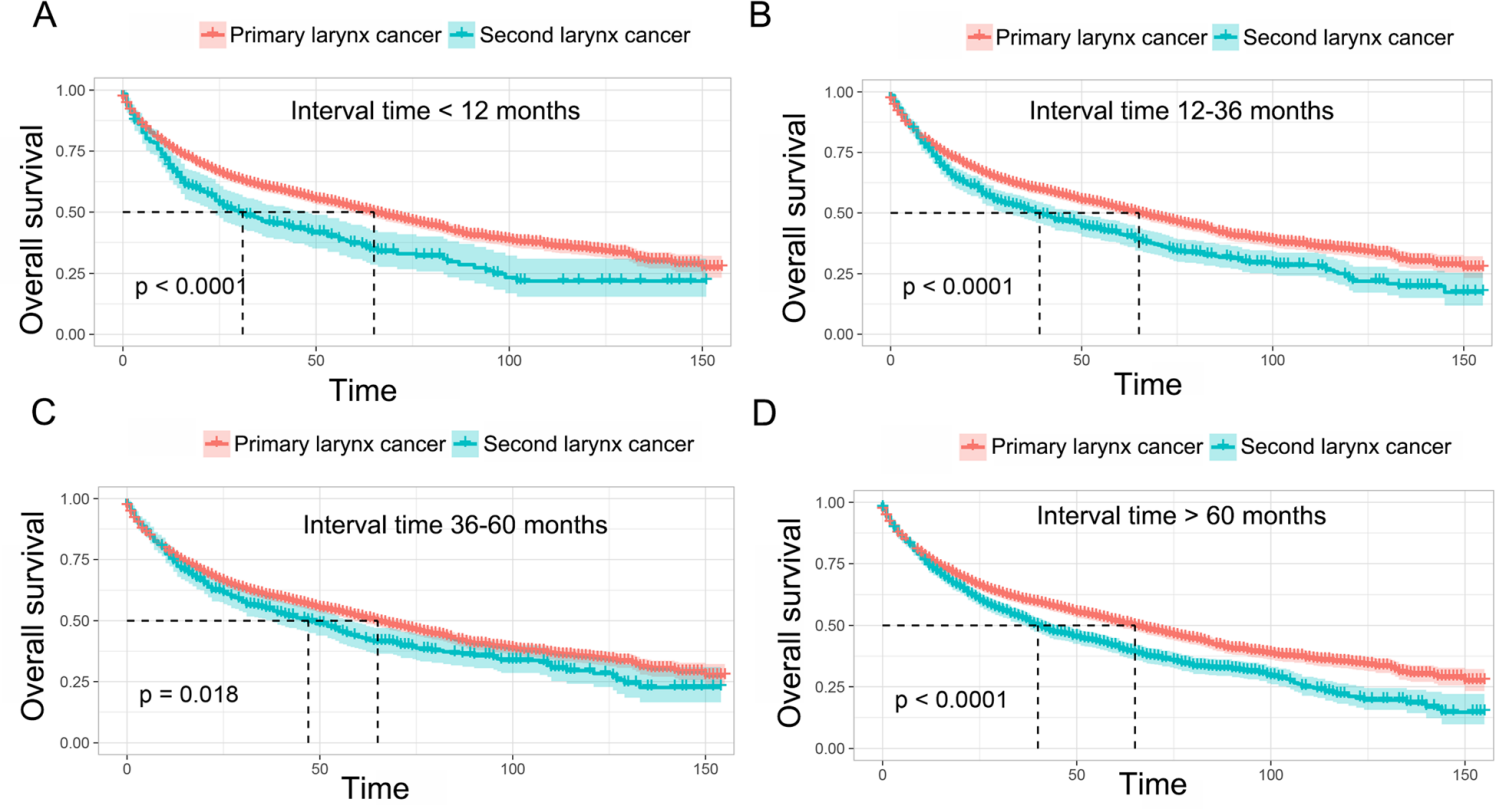
Kaplan-Meier survival curves of prior cancer impact on the overall survival (OS) stratified by timing of prior cancer in patients with larynx cancer. **a** The OS analysis with time interval less than 12 months; **b** The OS analysis with time interval between 12 and 36 months; **c** The OS analysis with time interval between 36 and 60 months; **d** The OS analysis with time interval longer than 60 months

**Fig. 4:**
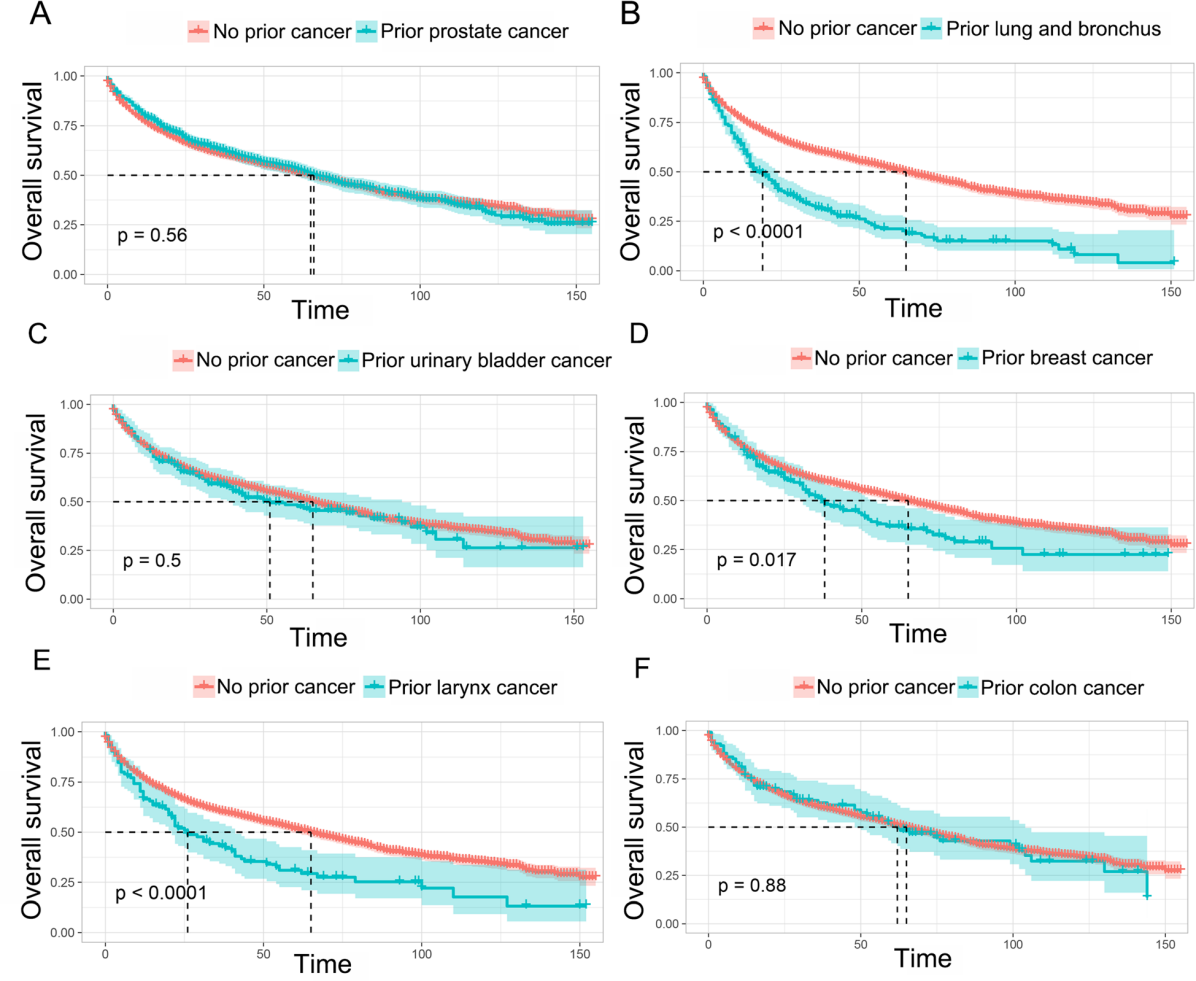
Kaplan-Meier survival curves of prior cancer impact on the overall survival (OS) stratified by different types of prior cancer in patients with larynx cancer. **a** The impact of prior prostate cancer on OS; **b** The impact of prior lung and bronchus cancer on OS; **c** The impact of prior urinary bladder cancer on OS; **d** The impact of prior breast cancer on OS; **e** The impact of prior larynx cancer on OS; **f** The impact of prior colon cancer on OS

As shown in Fig. 2a and Fig. S3A, patients with prior cancer history had significant inferior OS but decreased LCSM compared to those patients without prior cancer. After adjusted for propensity scores, a similar result was shown in Fig. 2b and Fig. S3B. Then, subgroup analyses were conducted for patients stratified by timing and types of prior cancer. Figure 3 indicated that patients with a prior cancer diagnosis had significant adverse impact on OS regardless of the time interval between the index larynx cancer and the prior cancer diagnosis. For LCSM, our results showed that patients with a prior cancer history had a marginal or significantly decreased LCSM compared to patients without a history of prior cancer, especially when the time interval was larger than 12 months (Fig. S4). As shown in Fig. 4 and Fig. S5, the impact of prior cancer on the OS and LCSM of larynx cancer patients varied extensively with different cancer types. The result showed that a prior prostate cancer diagnosis had no significant impact on the OS of larynx cancer patients, but exerted a significant protective factor for LCSM (Fig. 4a and Fig. S5A). On the contrary, larynx cancer patients with a prior lung or breast cancer diagnosis had dramatically inferior OS as compared to patients without a prior history of cancer, but not the LCSM (Fig. 4b and d, and Fig. S5B and D). Moreover, larynx cancer patients with a prior history of urinary bladder cancer or colon cancer both had similar OS and LCSM in comparison with patients without a prior cancer history (Fig. 4c and f, and Fig. S5C and F). Besides, a prior larynx cancer showed an inferior OS as well as increased LCSS, as compared to patients without a prior cancer diagnosis (Fig. 4e and Fig. S5E).

As shown in Table 4, the multivariate Cox analysis for patients’ OS indicated that a history of prior cancer was an independent risk factor for OS (AHR =1.30; 95% CI [1.21–1.41]). On the contrary, the multivariate competitive risk analysis showed that a history of prior cancer was an independent protective factor for LCSM (AHR = 0.83; 95% CI [0.72–0.94]). This data, combined with the OS result from the Cox analysis, suggested that patients with prior history of cancer were more likely to die from non-larynx cause than those without prior cancer. In addition, the Cox model suggested that a married status and treatment modalities including surgery, and radiotherapy were significant protective factors for OS of larynx patients with prior cancer history, whereas later AJCC stage, and high histological grade were risk factors. The competing risk model determined that a married status and treatment modalities like surgery and radiotherapy were protective factors for LCSM of patients with prior cancer history (Table 4).

**Table 4:** Comprehensive multivariable-adjusted Hazard Ratios for overall survival and larynx cancer-specific mortality

Characteristic	Overall survival		Larynx cancer-specific mortality	
	AHR (95% CI)	P value	AHR (95% CI)	P value
All patient after PSM				
Primary larynx cancer	1.0 (Ref)		1.0 (Ref)	
Second larynx cancer	1.30 (1.21–1.41)	<.001	0.83 (0.72–0.94)	0.004
Subgroup with prior cancer				
Age				
< 45	1.0 (Ref)		1.0 (Ref)	
45–64	1.12 (0.53–2.39)	0.765	2.98 (0.39–22.6)	0.290
> 64	1.60 (0.76–3.38)	0.216	3.86 (0.51–29.3)	0.190
Gender				
Male	1.0 (Ref)		1.0 (Ref)	
Female	0.89 (0.78–1.03)	0.111	0.85 (0.65–1.10)	0.210
Race				
White	1.0 (Ref)		1.0 (Ref)	
Black	0.99 (0.87–1.15)	0.986	1.15 (0.89–1.48)	0.290
Others/Unknown	0.81 (0.60–1.09)	0.164	1.43 (0.93–2.22)	0.110
Marital status				
Single	1.0 (Ref)		1.0 (Ref)	
Married	0.73 (0.62–0.85)	<.001	0.75 (0.56–0.98)	0.040
Other status ^a^	1.09 (0.92–1.29)	0.303	1.03 (0.77–1.40)	0.830
Unknown	0.79 (0.61–1.02)	0.072	0.59 (0.35–1.01)	0.053
AJCC stage				
I	1.0 (Ref)		1.0 (Ref)	
II	1.73 (1.46–2.05)	<.001	2.00 (1.39–2.88)	<.001
III	2.58 (2.19–3.05)	<.001	3.22 (2.29–4.54)	<.001
IV	3.95 (3.37–4.63)	<.001	5.23 (3.80–7.19)	<.001
Unknown	1.59 (1.28–1.97)	<.001	2.57 (1.70–3.88)	<.001
Histological grade				
Grade I	1.0 (Ref)		1.0 (Ref)	
Grade II	1.04 (0.87–1.24)	0.665	1.06 (0.75–1.50)	0.760
Grade III	1.19 (0.98–1.44)	0.085	1.05 (0.72–1.52)	0.820
Grade IV	2.42 (1.39–4.20)	0.002	0.94 (0.28–3.23)	0.920
Unknown	1.12 (0.93–1.35)	0.238	0.98 (0.67–1.41)	0.890
Surgery				
No surgery	1.0 (Ref)		1.0 (Ref)	
Surgery	0.57 (0.50–0.64)	<.001	0.68 (0.54–0.85)	<.001
Chemotherapy				
No chemotherapy	1.0 (Ref)		1.0 (Ref)	
Chemotherapy	0.82 (0.71–0.95)	0.006	0.94 (0.73–1.20)	0.610
Radiotherapy				
No radiation	1.0 (Ref)		1.0 (Ref)	
Radiation	0.56 (0.49–0.63)	<.001	0.67 (0.56–0.88)	0.002

*Abbreviations*: *AHR* adjusted hazard ratio, *Ref* reference category

Note: The multivariate analysis was adjusted for age, race, marital status, histologic grade, AJCC T stage, and treatment modalities (surgery, chemotherapy, and radiation)

^a^ other status including divorced, widowed, separated or domestic partner

**Fig. 5:**
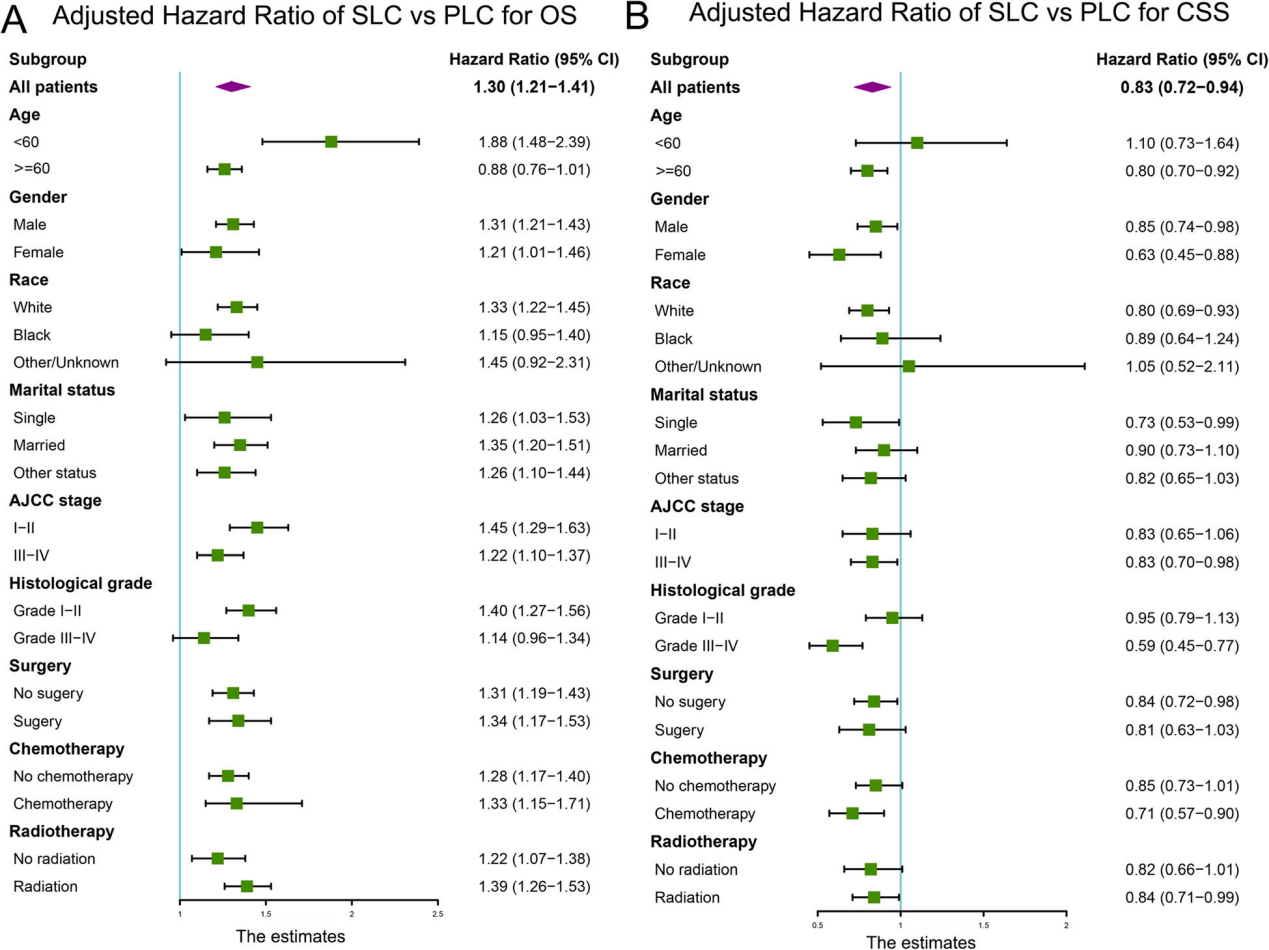
Forest plot of the adjusted hazard ratios (AHR) for survival of larynx cancer patients with or without prior cancer history. **a** The overall survival (OS) analysis; **b** The larynx cancer-specific mortality (CSM) analysis

Subgroup analyses were further performed to compare the OS and LCSM of larynx cancer patients with or without prior cancer history. As shown in Fig. 5a, a prior cancer history was significantly associated with shorter OS in most subgroups, except in patients with younger age (< 45), non-white race or higher histological grade (III-IV). However, regarding to LCSM, a prior cancer history tended to act as a protective factor in most subgroups (Fig. 5b).

## Discussion

The current study focused on the impact of a prior cancer history on clinical outcomes among patients with larynx cancer. The findings of this study may provide implications for the exclusion criteria of relevant clinical trials. In our study, 9.8% of patients had exactly one prior cancer before the diagnosis of larynx cancer. The median time interval between larynx cancer and prior cancers was 5.3 years in this cohort, longer than several previously reported cancers [13, 19]. Prostate cancer, lung and bronchial cancer, urinary bladder cancer and breast cancer were the most prevalent types of prior cancer in the larynx cohort, of which the histologic distribution was different from other cancer types, such as breast cancer [19], lung cancer [16] and nasopharyngeal cancer [13]. The high proportion of prior prostate or breast cancer history in our cohort and other reported cohort suggests its high prevalence in the US general population [7, 13, 16]. In addition, the lung and bronchial cancer or urinary cancer, such as bladder and kidney, were closely related to smoking, which was also the leading cause of larynx cancer [20].

In modern clinical practice, patients previously diagnosed with cancers are often excluded in most of clinical trials due to the assumption that a prior cancer might impact the survival outcomes. This stringent eligibility criteria might hinder clinical trial enrollment of a substantial number of patients, thus limiting generalizability, extending trial durations, and leading to a premature trial termination, especially amongst rare malignancies with low incidence [14, 21]. Recently, several studies have reported that a prior cancer history might not adversely affect patients’ survival who suffered a subsequent cancer. For example, a previous retrospective study by Laccetti et al. reported that a prior cancer history did not adversely affect clinical outcomes of patients with advanced lung cancer, regardless of the different stages or types of prior cancer [16]. Another study also suggested that the prognosis of patients with uterine papillary serous carcinoma was not affected by prior breast cancer and tamoxifen exposure [17]. However, the impact of a prior cancer history on patients with larynx cancer has yet to be extensively studied.

In the present study, we found that a prior cancer history adversely impacted the overall survival of larynx cancer patients but seemed to be associated with a decreased larynx cancer-specific mortality. Interestingly, when stratified by timing of prior cancer, we found that the positive effect on larynx cancer-specific mortality was gradually increased when the time interval was longer than 12 months. This could be potentially explained by the early diagnosis due to regular follow-up examinations or reduced exposure to risk factors such as tobacco. Our results also indicated that the impact of prior cancer on the OS and LCSM of larynx cancer patients varies distinctly across different cancer types. An inferior overall survival was observed in larynx cancer survivors with prior lung, breast or larynx cancer, but not prostate, bladder or colon cancers. In addition, a possible decreased larynx cancer-specific mortality was observed within prostate cancer survivors. Hence, it seems cancers with excellent prognoses, such as prostate or bladder cancer, had no substantial impact on the clinical outcome of subsequent larynx cancer, primarily because of its indolent course, early diagnosis, and favorable response to local treatment [16]. These findings inspired us that certain types of prior cancer could be considered in clinical trials regarding to larynx cancer patients.

The multivariate Cox analysis indicated that patients with prior cancer had inferior overall survival than those without prior cancer. Subsequently, the subgroup analyses further indicated that only patients with younger age (< 45), non-Caucasians or higher histological grade (III-IV) did not have inferior survival as compared to patients with no prior cancer. Those results were, however, not consistent with several studies reporting a non-adverse or even positive impact a prior cancer history has on the OS amongst patients with other types of malignancies [16, 22–24]. Together with the subgroup analysis based on the timing and types of prior cancer, larynx cancer patients with prior cancers should be prudently considered for clinical trials.

Interestingly, the multivariate Fine and Gray’s regression analysis showed a decreased larynx cancer-specific mortality in patients with prior cancer as compared to those without a prior history of cancer. This result echoes earlier findings concentrating on other site [13, 25, 26], indicating that patients with prior history of cancer had a dramatically increased risk for death from prior cancer or other non-cancer cause compared to those without prior cancer. These risks should be carefully considered in the treatment toward subsequent larynx cancer occurring in such patients with prior history of cancer. The subgroup analysis further found that a prior cancer history could exhibit a protective factors for larynx cancer-specific mortality especially in patients with older age, white race, single status, later AJCC stage, higher histological grade, receiving no surgery, and receiving chemotherapy or radiotherapy. Potential explanations includes individualized patient’s biology and treatment response, or more frequent engagement in healthcare systems.

Previous epidemiological data highlighted large racial disparities in survival of patients with many cancer types [27, 28], which might due to many confounding socioeconomical and behavioral factors, such as diet, smoking, alcohol abuse, and access to screening and treatment. Inconsistently, no survival difference was observed among patients with different race in our study. Moreover, various studies yielded conflicting results about the impact of marital status on survival of cancer patients, with protective [29, 30], mixed [31, 32], or nonsignificant [33, 34] effects identified. Most of these investigations involved a single malignancy. In our study, as supplementary, both the multivariate Cox survival analysis and the Fine and Gray’s regression analysis showed that a married status served as a protective factor for prognosis of patients with prior cancer history. Either financial or psychological support from family could be important contributing factors.

There are several limitations to our study. Firstly, some valuable information such as comorbidities and the toxicity of treatment on prior cancer were unavailable in the SEER database. Secondly, our study is limited by the intrinsic weaknesses of retrospective databases wherein selection bias is inherent. We believe that all the observed results in our study should be prospectively validated.

## Conclusion

In summary, patients with prior cancer history had inferior overall survival but decreased larynx cancer-specific mortality in comparison with those without prior cancer. These impacts varied across patients with different timing, prior cancer types and clinicopathologic features. Except for a few particular prior cancer, clinical trials should be considered prudently for laryngeal cancer patients with prior cancers.

## Supplementary Information

**Additional file 1:** **Figure S1.** The flowchart of case selection.

**Additional file 2:** **Figure S2.** Histogram of standardized differences before and after PSM. (A) Raw larynx cancer patients with prior cancer; (B) Matched larynx cancer patients with prior cancer; (C) Raw larynx cancer patients without prior cancer; (B) Matched larynx cancer patients without prior cancer.

**Additional file 3:** **Figure S3.** Cumulative incidence curves of prior cancer impact on the larynx cancer-specific mortality (LCSM) of larynx cancer patients with or without prior cancer. (A) The LCSM analysis before Propensity score matching (PSM); (B) The LCSM analysis after PSM. The solid line represents LCSM and the dotted line represents non-LCSM.

**Additional file 4:** **Figure S4.** Cumulative incidence curves of larynx cancer-specific mortality (LCSM) of larynx cancer patients stratified by timing of prior cancer. (A) The LCSM analysis with time interval less than 12 months; (B) The LCSM analysis with time interval between 12 and 36 months; (C) The LCSM analysis with time interval between 36 and 60 months; (D) The LCSM analysis with time interval longer than 60 months. The solid line represents LCSM and the dotted line represents non-LCSM.

**Additional file 5:** **Figure S5.** Cumulative incidence curves of larynx cancer-specific mortality (LCSM) of larynx cancer patients stratified by different types of prior cancer. (A) The impact of prior prostate cancer on LCSM; (B) The impact of prior lung and bronchus cancer on LCSM; (C) The impact of prior urinary bladder cancer on LCSM; (D) The impact of prior breast cancer on LCSM; (E) The impact of prior larynx cancer on LCSM; (F) The impact of prior colon cancer on LCSM. The solid line represents LCSM and the dotted line represents non-LCSM.

**Additional file 6.**

## Data Availability

The datasets generated and/or analysed during the current study are available in the Surveillance, Epidemiology, and End Results Program repository, https://seer.cancer.gov/data/.

## Abbreviations

SEER: Surveillance, Epidemiology, and End Results
OS: overall survival
CSS: cancer-specific survival
PSM: Propensity score matching
AHR: Adjusted hazard ratio
IQR: Interquartile range
