# Supplementary figures and images for: Impact of prior cancer history on the survival of patients with larynx cancer
Source: BMC Cancer. 2020 Nov 23;20:1137. doi: 10.1186/s12885-020-07634-2 (PMC7685658; doi:10.1186/s12885-020-07634-2)

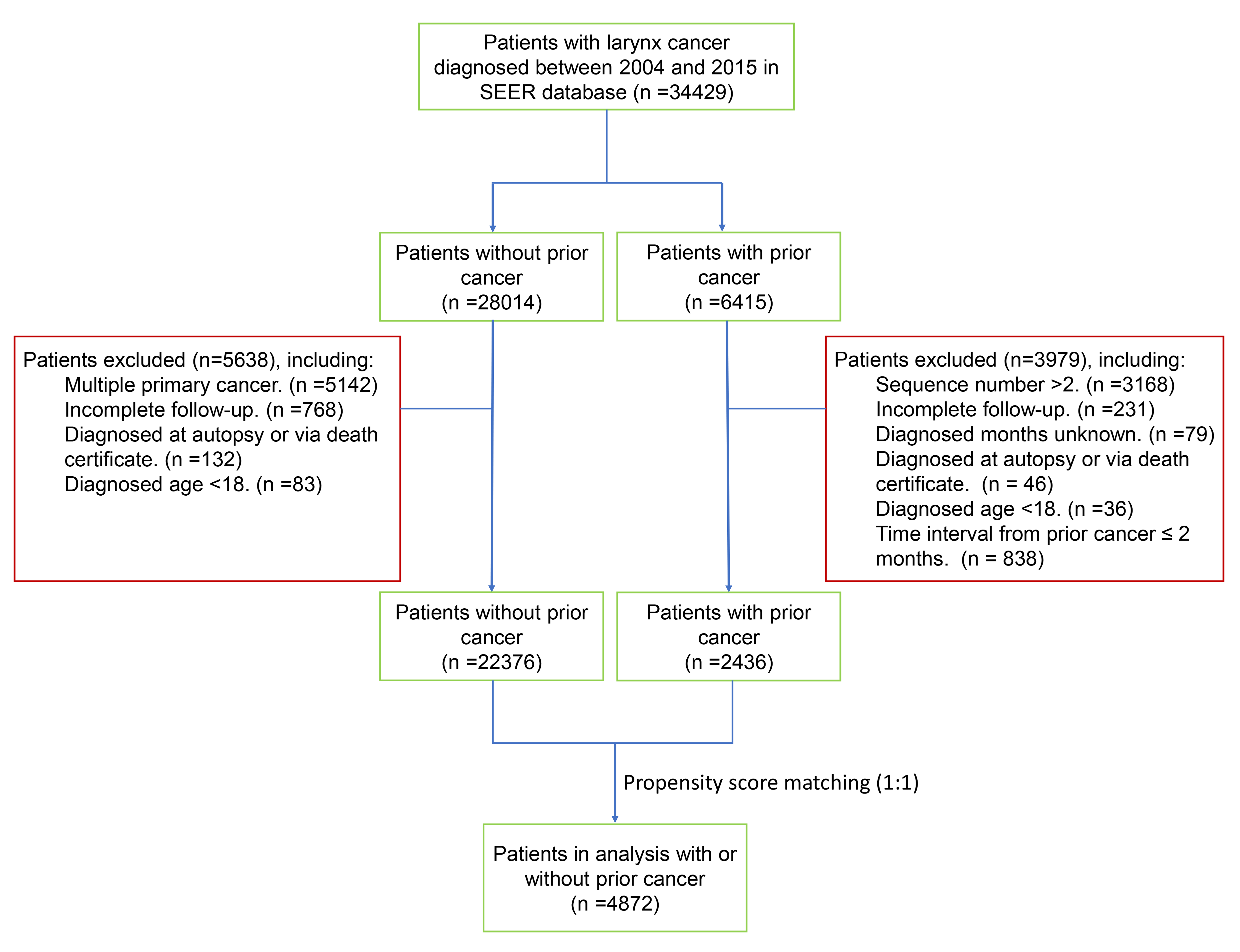

Supplement: Supplementary file 1 — Additional file 1: Figure S1. The flowchart of case selection. [file 12885_2020_7634_MOESM1_ESM.tif]

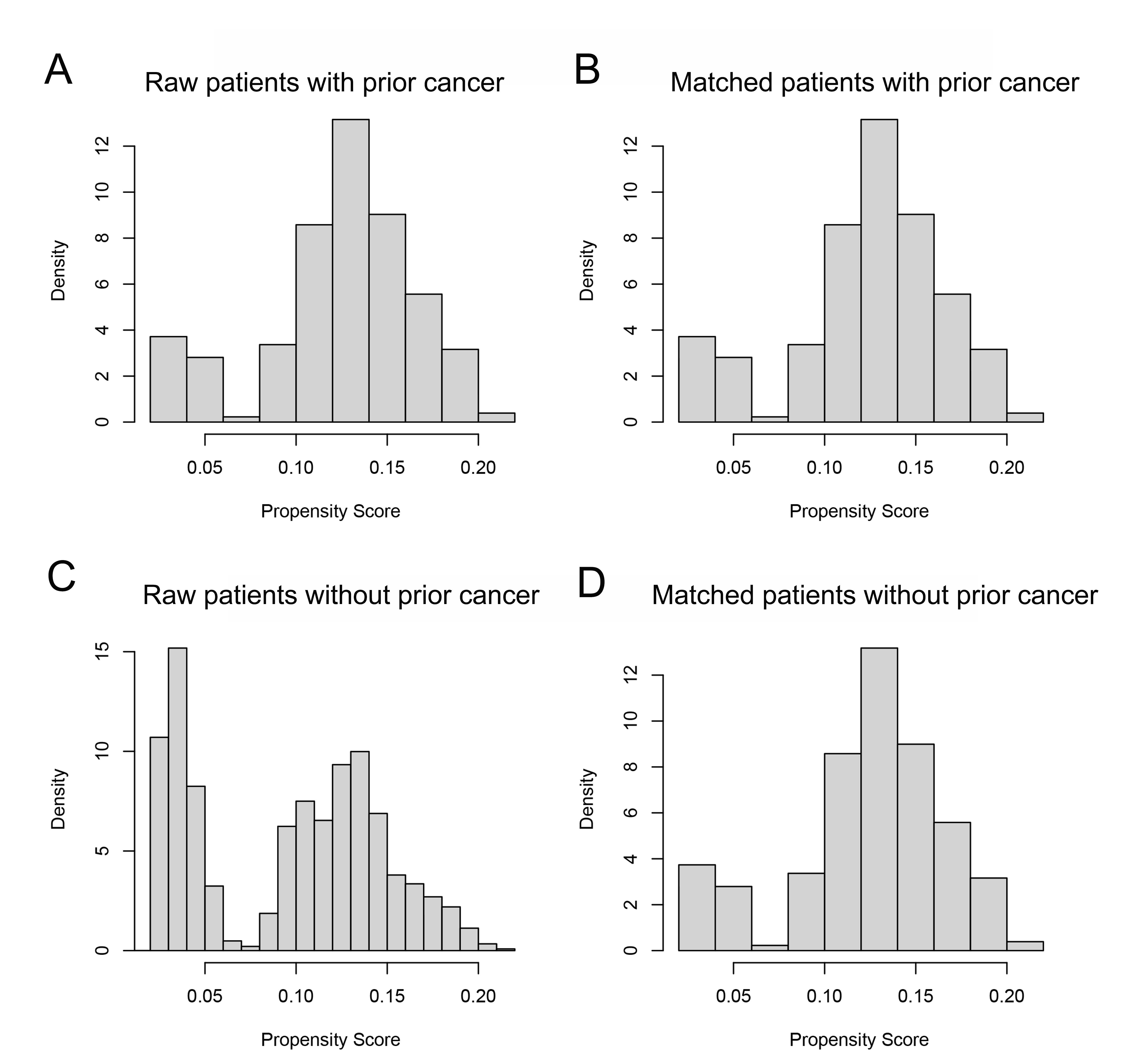

Supplement: Supplementary file 2 — Additional file 2: Figure S2. Histogram of standardized differences before and after PSM. (A) Raw larynx cancer patients with prior cancer; (B) Matched larynx cancer patients with prior cancer; (C) Raw larynx cancer patients without prior cancer; (B) Matched larynx cancer patients without prior cancer. [file 12885_2020_7634_MOESM2_ESM.tif]

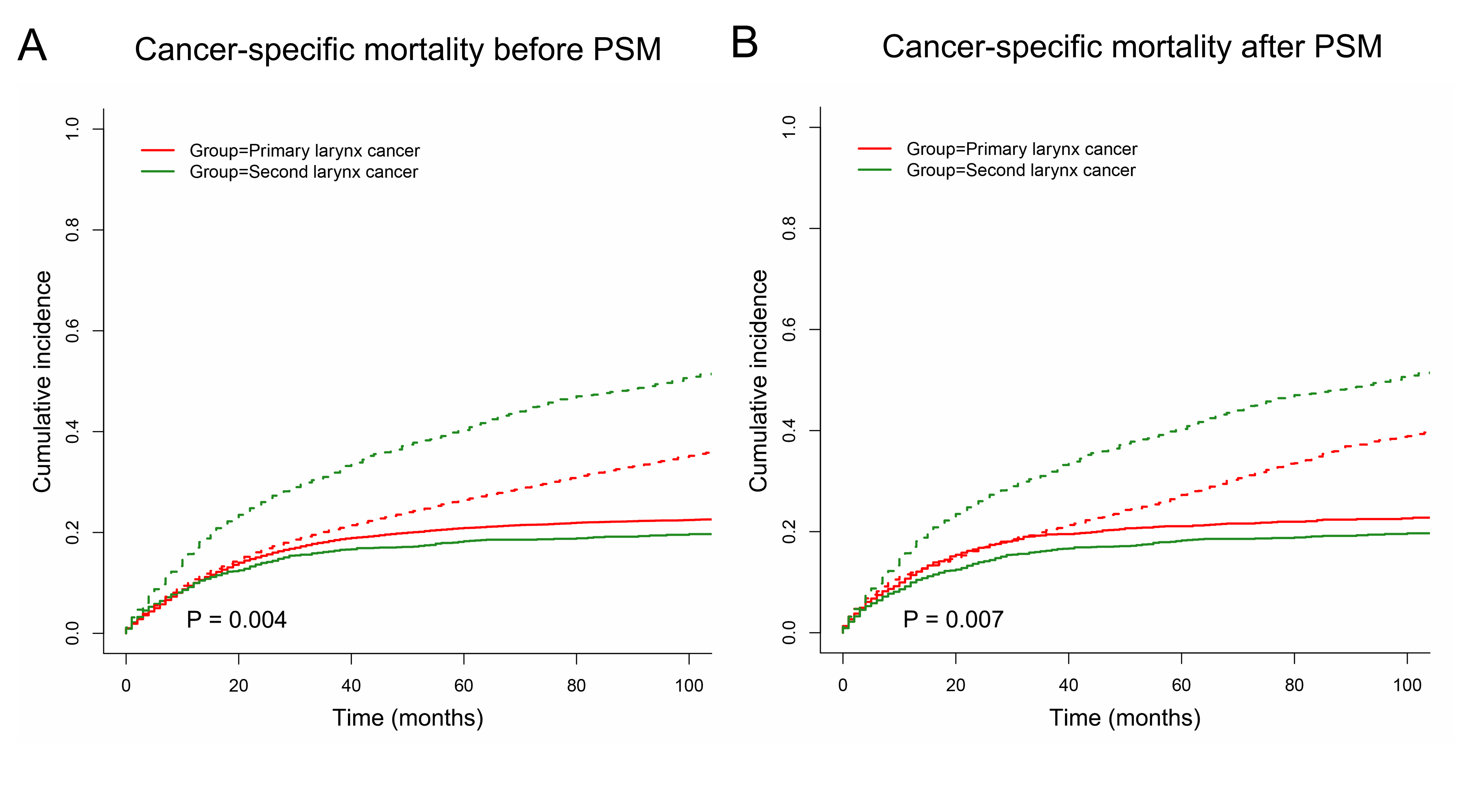

Supplement: Supplementary file 3 — Additional file 3: Figure S3. Cumulative incidence curves of prior cancer impact on the larynx cancer-specific mortality (LCSM) of larynx cancer patients with or without prior cancer. (A) The LCSM analysis before Propensity score matching (PSM); (B) The LCSM analysis after PSM. The solid line represents LCSM and the dotted line represents non-LCSM. [file 12885_2020_7634_MOESM3_ESM.tif]

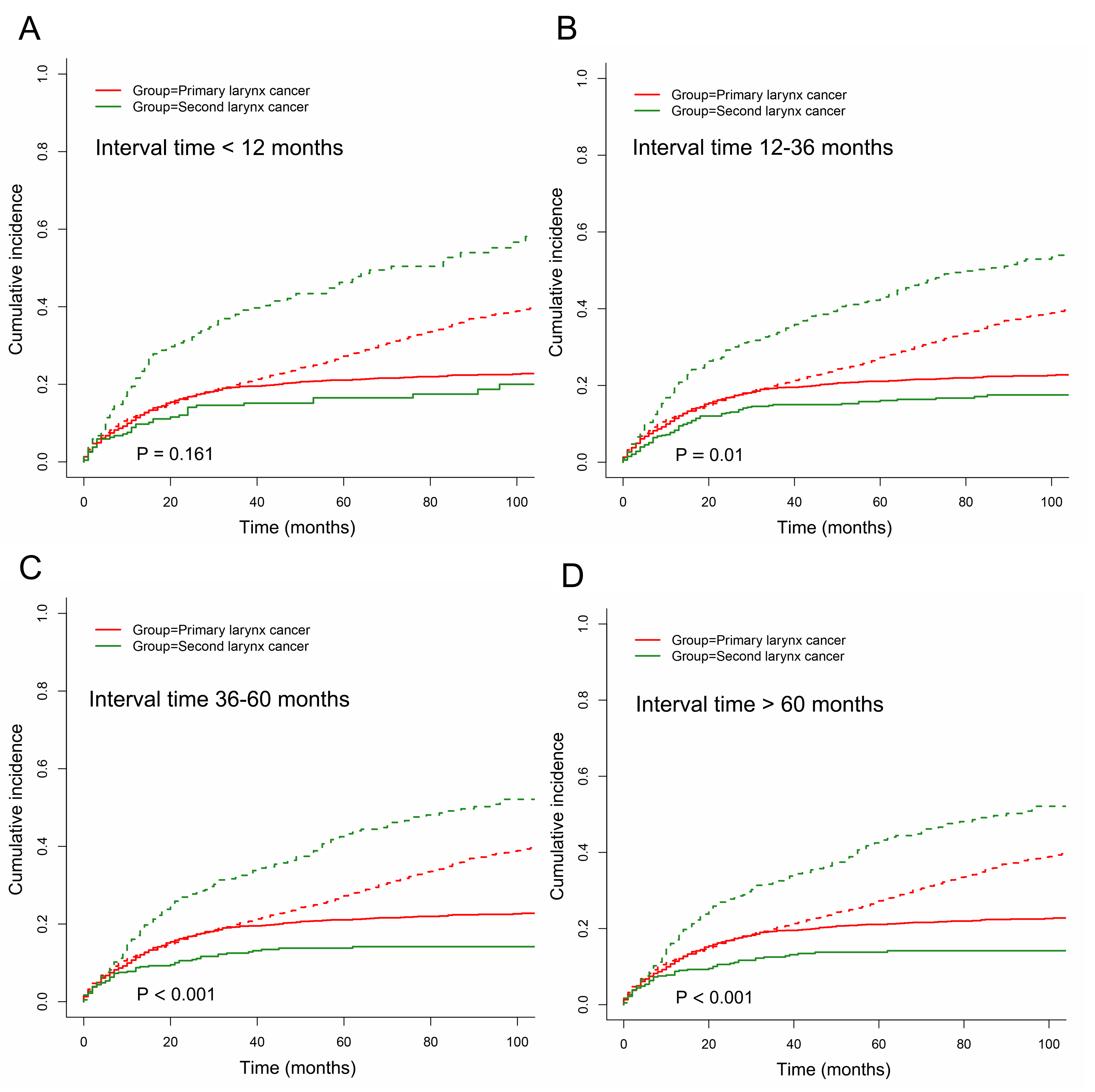

Supplement: Supplementary file 4 — Additional file 4: Figure S4. Cumulative incidence curves of larynx cancer-specific mortality (LCSM) of larynx cancer patients stratified by timing of prior cancer. (A) The LCSM analysis with time interval less than 12 months; (B) The LCSM analysis with time interval between 12 and 36 months; (C) The LCSM analysis with time interval between 36 and 60 months; (D) The LCSM analysis with time interval longer than 60 months. The solid line represents LCSM and the dotted line represents non-LCSM. [file 12885_2020_7634_MOESM4_ESM.tif]

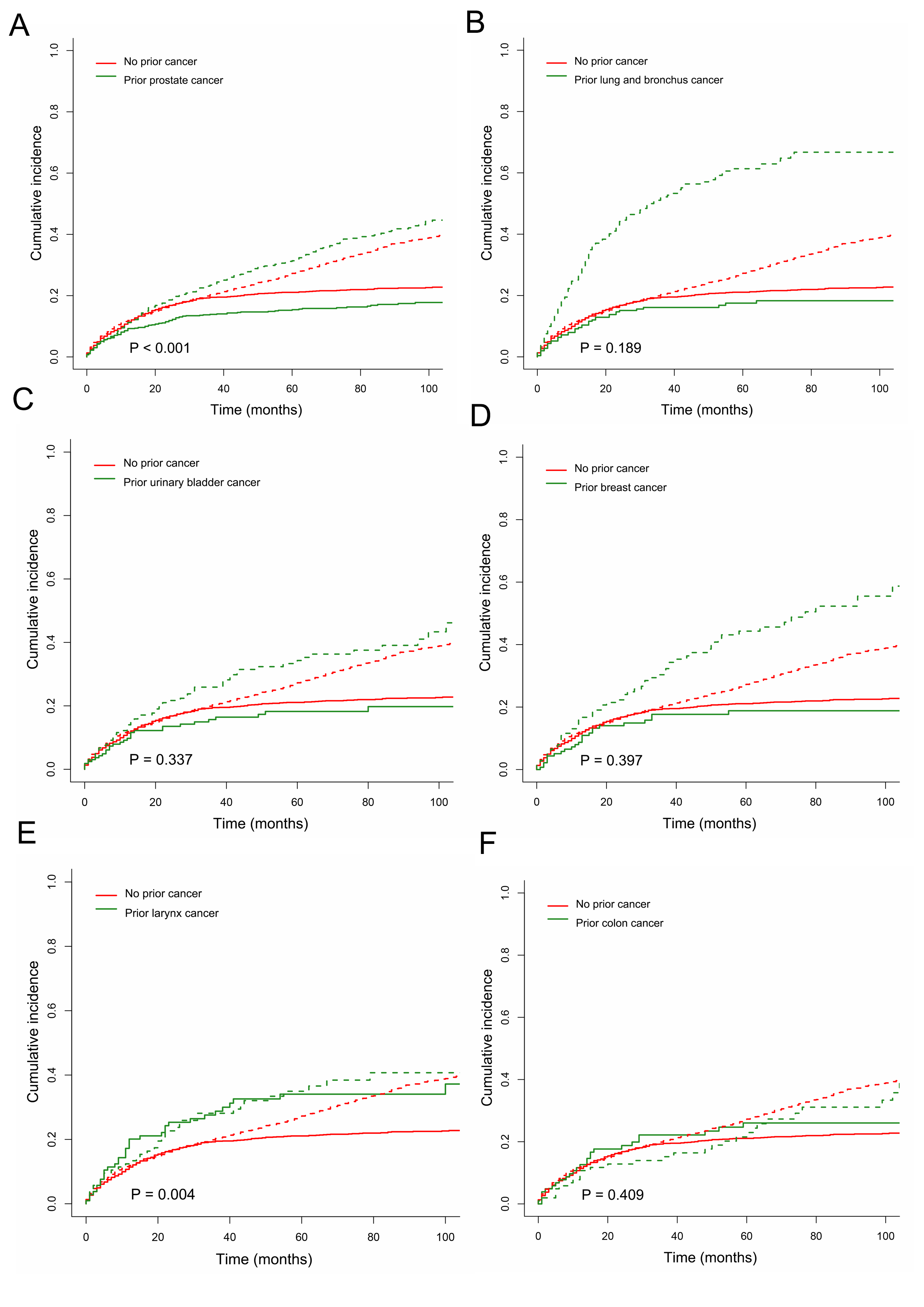

Supplement: Supplementary file 5 — Additional file 5: Figure S5. Cumulative incidence curves of larynx cancer-specific mortality (LCSM) of larynx cancer patients stratified by different types of prior cancer. (A) The impact of prior prostate cancer on LCSM; (B) The impact of prior lung and bronchus cancer on LCSM; (C) The impact of prior urinary bladder cancer on LCSM; (D) The impact of prior breast cancer on LCSM; (E) The impact of prior larynx cancer on LCSM; (F) The impact of prior colon cancer on LCSM. The solid line represents LCSM and the dotted line represents non-LCSM. [file 12885_2020_7634_MOESM5_ESM.tif]
