# Supplementary material for: Impact of prior cancer history on the survival of patients with larynx cancer
Source: BMC Cancer. 2020 Nov 23;20:1137. doi: 10.1186/s12885-020-07634-2 (PMC7685658; doi:10.1186/s12885-020-07634-2)
Supplement: Supplementary file 6 — Additional file 6. [file 12885_2020_7634_MOESM6_ESM.docx]

**Supplementary material for methods**

**PSM analysis**

In order to minimize the baseline confounders, a one-to-one PSM between larynx cancer patients with or without prior cancer was conducted by utilizing the nearest-neighbor matching method with a caliper distance of 0.2. Based on the result of Pearson chi-square tests, we included all the covariates in patients’ baseline characteristics into the PSM algorithm, including year of diagnose, age, race, gender, marital status, histological grade, American Joint Committee on Cancer (AJCC, sixth edition) stage, treatment modalities (surgery, chemotherapy and chemotherapy). A histogram of standardized differences before and after PSM was plotted to visually exhibit the properties of the matching procedure (**Fig. S2**). Besides, the propensity-score adjusted p-value was provided as well. The PSM procedure and the calculation of standardized differences were performed using R packages of *MatchIt*.
